# Manual of the Diseases of Children

**Published:** 1898-12

**Authors:** 


Manual of the Diseases of Children. By John Madison Taylor, A. M., M. D., Professor of Diseases of Children, Philadelphia Polyclinic; Assistant Physician to the Children's Hospital and to the Orthopedic Hospital; Consulting Physician to the Elwyn and the Vineland Training Schools for Feeble-Minded Children; Neurologist to the Howard Hospital, etc.; and William H. Wells, M. D., Adjunct Professor of Obstetrics and Diseases of Infancy in the Philadelphia Polyclinic; late Assistant Demonstrator of Clinical Obstetrics and Diseases of Infancy in Jefferson Medical College. With 60 illustrations. 650 pages. Philadelphia: P. Blakiston's Son & Co. 1898.

One takes up the work of Taylor and Wells with the feeling that it has an excuse for being written, in that such a book as it aims to be, a "brief but competent guide for the student and practitioner," is needed. Yet in reading it there grows upon one the regret that a book so well conceived and in some respects so well executed, should be marred so needlessly. The book covers the field of pediatrics as completely as a work of its kind can be expected to, laying particular stress upon treatment, which subject in the main is very satisfactorily handled. The manual in part is a compilation, and its most noticeable defects are due to haste or carelessness, or both, on the part of the compilers. For instance, a table giving the percentage composition of cow's milk is quoted from Rotch, the all-important proteid percentage being omitted. Again, the chapter devoted to milk modification appears in the index with the rather unsatisfactory title of the breeds of cows best adapted for infant feeding. To be sure these are trifling errors, but they are unnecessary. In diseases of the stomach the authors fail to make a distinction between acute gastritis and acute gastric indigestion, though on the whole the chapter on the diseases of the digestive organs is good. The paragraphs devoted to the care of the premature infant might better have been left out, being both inadequate and unscientific.

The authors are at their best when original, the chapter on physical development, in which the work of the compiler is not in evidence, being the best in the book and well worth reading. The book has sufficient merit to warrant the prophecy of a second edition, from which the blemishes which mar the pages of the present shall be eliminated. M. J. F.

